# Consumer Demand for Milk and the Informal Dairy Sector Amidst COVID-19 in Nairobi, Kenya

**DOI:** 10.1016/j.cdnut.2023.100058

**Published:** 2023-02-25

**Authors:** Silvia Alonso, Moira Donahue Angel, Emmanuel Muunda, Emily Kilonzi, Giordano Palloni, Delia Grace, Jef L. Leroy

**Affiliations:** 1 Animal and Human Health Program, International Livestock Research Institute, Addis Ababa, Ethiopia; 2 Poverty, Health and Nutrition Division, International Food Policy Research Institute, Washington DC, United States; 3 Animal and Human Health Program, International Livestock Research Institute, Nairobi, Kenya; 4 Food and Markets Department, Natural Resources Institute, University of Greenwich, United Kingdom

**Keywords:** dairy, informal sector, milk consumption, children, COVID-19, Kenya, milk vendors, milk sales

## Abstract

**Background:**

The coronavirus disease 2019 (COVID-19) pandemic has had large negative effects on countries’ economies and individual well-being throughout the world, particularly in low- and middle-income countries. Pandemic-related changes in behavior and government restrictions in Kenya may have negatively affected food supply chains and household food access; however, the empirical evidence is currently limited.

**Objectives:**

The study explored changes in informal milk markets, dairy consumption, and food insecurity among low-income households in urban and periurban Nairobi, Kenya, following the start of the COVID-19 pandemic in the country.

**Methods:**

Baseline data on milk sales and consumption were collected in late 2019 from dairy vendors operating in the informal sector and their dairy customers. We conducted 2 longitudinal telephone surveys with the same study participants in July and September–October 2020, respectively.

**Results:**

At the first follow-up, the volume of milk sold by informal vendors had dropped by 30% compared with their baseline level, and the volume of milk from informal markets consumed by households decreased by 23%. By the second follow-up, the volume of milk sold and consumed had recovered somewhat but remained lower than the volume observed 1 y prior in the same season. Large reductions in the consumption of other animal–sourced products were also observed. The rate of food insecurity increased by 16 and 11 percentage points in the first and second follow-up periods, respectively, compared with baseline.

**Conclusions:**

The evidence, therefore, suggests that the timing of the pandemic and the related restrictions were associated with a decrease in the supply and consumption of milk from informal markets in Nairobi and a decrease in the food security of periurban consumers.

## Introduction

In the span of its first 2 y, the COVID-19 pandemic caused 450 million confirmed cases globally and led to nearly 6 million official death counts worldwide, although its true death toll could be 2–4 times higher [1,2]. Apart from direct impacts on morbidity and mortality, the pandemic also had large negative effects on countries’ economic outcomes and individual well-being. In 2020, an additional 97 million people fell into poverty, a “historically unprecedented increase in global poverty” [3]. Although increases in poverty were likely more severe during the early stages of the pandemic when measures such as lockdowns and curfews were common, projections suggest that in the poorest countries—including most of sub-Saharan Africa—the impact of COVID-19 on poverty persists and could be worsening [4].

Along with an increase in poverty, pandemic-driven changes in individual food purchasing and consumption behaviors and government-imposed restrictions aimed at containing the spread of the virus have negatively affected food security in many countries [5,6]. Early models predicted that COVID-19 disruptions to food supply chains would be greatest in post-farm operations and businesses, primarily in urban and periurban areas and for households involved in informal markets. These markets are characterized by traditional small businesses operating with limited infrastructure and are the markets where most people, especially the poor, buy their foods in low- and middle-income countries [7]. Accelerating food price increases from already rapid pre–COVID-19 trend [8] are most acutely felt by the vulnerable and poorest households in low- and middle-income countries, who spend a larger proportion of their budget on food [9]. When combined with reduced incomes, higher retail prices lead more households to have difficulty obtaining nutrient-adequate diets [10].

Kenya was one of the countries in Africa that responded promptly to the pandemic. Starting in March 2020, when the first COVID-19 case was reported in the country, the government established various measures to contain the spread. Closure of schools and commercial premises selling food (eateries) and international travel restrictions were in effect for most of 2020. A curfew from 19:00 to 05:00 (later relaxed to 22:00 to 04:00) was in place between March 2020 and October 2021. In Nairobi and a few other counties that experienced a higher number of COVID-19 cases, even more stringent restrictions were implemented, including a more strictly enforced curfew and travel restrictions. These restrictions are likely to have affected the supply of food to the city. Considering that most fresh food sold in Nairobi markets arrives daily from nearby counties where production is primarily concentrated, often moving at night and arriving in the early morning hours, curfews and travel restrictions are likely to have affected food supply. Furthermore, the closure of hotels and eateries in the evenings could have reduced the overall food demand and may, thus, have further reduced the supply of food to the city.

The COVID-related changes in behavior and government restrictions in Kenya may have negatively affected food supply chains and household food access; however, the empirical evidence is currently limited. Our primary objective was to understand how food system operations and household food access changed during this time. More specifically, we assessed purchases and sales by dairy vendors from the informal sector, as well as household food (including milk) purchases and consumption and household food security before and after the onset of the pandemic. We took advantage of the MoreMilk project, an ongoing study on the role of informal dairy markets on health and nutrition in periurban Nairobi. Data from the comprehensive in-person baseline survey data conducted in 2019 before the start of the pandemic were supplemented with longitudinal follow-up phone surveys in 2020.

Milk is a highly consumed food of animal origin in Kenya, and ∼70%–80% of the milk marketed in the country is sold through informal markets [11,12]. Per capita, milk consumption stands at ∼110 L per annum, well above that of other African countries [11,13]. And whereas the estimated *household* price elasticity of milk demand (that is, the change in consumption of milk in relation to the change in price) in Kenya is low [14], an increase in milk prices has been shown to reduce the household allocation of milk to young children [15]. By virtue of the low-price elasticity, increased prices may negatively affect the household resources available to purchase other key sources of nutrients. We used our data to study how sensitive the supply and consumption of milk are to shocks such as the COVID-19–related disruptions, and we investigated whether there were broader implications for household food consumption and household food security.

## Methods

### Study population

The MoreMilk survey was conducted in 3 periurban subcounties of Nairobi beginning in October 2019: Dagoretti North, Dagoretti South, and Kasarani. The survey was the baseline assessment for a randomized-controlled trial (RCT) that was stopped due to COVID-19 (clinicaltrials.gov registration: NCT04109521). The objective of the trial was to assess the impact of training, certification, and marketing intervention for milk vendors in the informal sector on milk safety and nutrition outcomes in children in urban and periurban areas of Kenya. The study participants were dairy vendors and their client households. The inclusion criteria for dairy vendors included selling unpacked liquid milk (that is, milk that had not gone through an industrial pasteurization and packaging process, commonly referred to as “milk from the informal market”) ≥5 d/wk to individual customer, having the intention to remain in the business for ≥12 mo; and operating a milkbar (that is, exclusively licensed to sell dairy products and eggs) or a shop/kiosk (that is, licensed to sell food and nonfood products), or being a street vendor (that is, not selling in a fixed building structure but selling from a fixed location along the street). Middlemen (individuals that purchase and transport milk from farms to distribute to businesses) and vendors catering exclusively to other commercial establishments were excluded, as were mobile street vendors (that is, selling from a mobile premise like a cart, motorbike, etc.) and vendors operating a milk dispenser (also called a milk-ATM) or selling exclusively at farm gate. These criteria were designed to ensure participants were representative of the type of vendors that sell most milk in periurban Nairobi and, considering the high turnaround of dairy businesses in the city, maximize the likelihood that participants recruited in the study will remain in business for the duration of the study. The household inclusion criteria were purchasing unpacked milk, obtaining ≥50% of the weekly milk supply from a vendor recruited in the study, and having ≥1 child between 12 and 48 mo of age at the time of recruitment. This age range was chosen to avoid promoting the consumption of cow milk in children under 1 y of age and to ensure that children would be <5 y of age at follow-up.

### Sampling and recruitment

The sample size at baseline was determined by the requirements of the RCT, which was stopped due to COVID-19. Our objective was to recruit 240 vendors and 852 households.

A geo-referenced census of dairy vendors was conducted in January 2019 to construct the sampling frame for the MoreMilk project. To meet the needs of this project, clusters of 1–5 eligible vendors were created such that all vendors in a cluster were located no further than 250 m apart from each other, and vendors in one cluster were located ≥250 m away from the nearest vendor in a different cluster [16]. Sampled vendors were visited and invited to participate. Client households were recruited from the participating vendors using 2 strategies. First, consenting vendors were given a leaflet to be displayed at the point of sale, explaining the study in simple terms to customers. Customers expressing interest in the study wrote down their names and telephone numbers on a registration sheet. When a vendor had successfully registered ≥10 households, the registration sheets were collected by the research team to initiate the recruitment of households. Second, if a vendor failed to register 10 households and agreed to accept support from the research team, an enumerator spent a few hours at the business approaching dairy customers, presenting the study, and registering interested households. For each vendor cluster, the complete list of registered households was randomly ordered, and households were called sequentially to check for eligibility and interest to participate in the study until a maximum of 7 households were recruited in each cluster. All vendors and households who participated in the baseline surveys were invited to participate in the 2 follow-up phone surveys.

Written informed consent was obtained from the business owner and from the primary household respondent before the start of the baseline interview. Verbal informed consent was obtained before each of the phone surveys. Consenting to participate in the baseline survey implied agreement to participate in all phases of the RCT: for dairy vendors, baseline, midterm, and endline surveys, and all the components of the MoreMilk trial; for households, baseline, and endline surveys. Participants were informed at baseline that the research team would publish the data collected from this project and that their data would be shared with relevant stakeholders; at follow-up, participants were reminded of the intent to share results of data collected and assured that their individual involvement in the project would be kept confidential. Vendors were not compensated in any form for their participation in the study with the understanding that receiving the project intervention provided a direct benefit to participants. Households received a bar of soap and 2 kg of maize flour at the time of the baseline survey as a token of appreciation for their time. Participants who participated in the follow-up phone surveys received 50 KSH (equivalent to 0.5 United States Dollar [USD]) of airtime. Results of the study were not shared with the individual participants. Confidentiality of data was assured by keeping datasets with identifiable information in a secure drive hosted by the International Livestock Research Institute, which was accessible only to the research team. Data analysis was performed exclusively with anonymized datasets.

### Timing

Baseline data were collected from October 2019 to January 2020 through an in-person survey. The follow-up phone surveys took place in July 2020 and between September 2020 and October 2020. The first case of COVID-19 was confirmed in Kenya on March 12, 2020. At the time of the first follow-up survey, a nationwide curfew from 19:00 to 05:00 was in place, there was a ban on social gatherings and bars, restaurants, and eateries was required to close by 19:00; places of worship were only allowed to operate under strict guidelines, and a previously imposed ban on travel to and from Nairobi had been lifted. At the time of the second follow-up survey, most of the major COVID-19–related restrictions had been lifted: curfew hours had been adjusted to 23:00 to 04:00, restaurants and eateries were allowed to operate until 22:00, and schools reopened for some classes.

### Data collection

Questionnaires were administered in Kiswahili by extensively trained and closely supervised fieldworkers. Training included the project aim and scope, understanding the survey questions, standardization of asking questions across field workers, and ethical aspects of research (that is, data collector and participant relation, administering and obtaining informed consent, and confidentiality). The training also included pilot testing of the tools with vendors and households outside of the study area. Around 25 fieldworkers conducted interviews at baseline, and 8 fieldworkers conducted phone interviews.

#### *Vendor survey*

The MoreMilk baseline survey collected information on vendor demographic and socioeconomic characteristics, on business profiles with emphasis on the dairy-focused part of the business (that is, type of business, ownership arrangements, and customer base), dairy sourcing, handling and hygiene of dairy products, business revenue and expenses (general and dairy-specific), business practices (record keeping, stock control, marketing, financial planning, and price setting), credit and savings, business capital, and capacity development. The phone surveys took ∼20 min to be administered and collected information on whether the business was still operating, dairy sourcing, volumes of dairy sold, prices, and business profits.

#### ***Household surve****y*

At baseline, data were collected on household demographic and socioeconomic characteristics, food and nonfood consumption and expenditure (including dairy consumption and expenditure), and household food insecurity. In addition, we queried mothers about the health of their children, their knowledge of child feeding practices, and usual milk handling practices. The 2 follow-up mobile phone surveys were considerably shorter and included a limited set of questions on household composition, household food and nonfood consumption and expenditure for select commodities (including dairy), and household food insecurity. Each phone survey took ∼20 min to complete.

At baseline, household consumption and expenditure data were collected for a list of 40 food (14 dairy and 26 nondairy) and 33 nonfood items. Based on the 2015–2016 Kenyan Household Budget Survey (KHBS), these items comprised ∼80% of total expenditure for households in Nairobi, on average. The list of food and nonfood items was shortened for the follow-up mobile phone survey to 6 nondairy food items, 7 dairy items, and 8 nonfood items. We asked about the number of foods consumed and purchased by the household in the last 7 d. The dairy food module asked only about dairy purchases. We assumed, based on the perishability of dairy products, that the dairy quantities purchased were the same quantities consumed by the household. Household food insecurity was assessed using the household food insecurity access scale (HFIAS) [17].

#### *Outcome* variables

##### Vendor

For each dairy product sold by the vendor, the amounts purchased and sold were converted as needed to liters using conversion factors from the FAO/International Network of Food Data Systems (FAO/INFOODS) Density Database Version 2.0 [18]. Volumes of dairy products purchased and sold in the past 7 d were winsorized at the 97.5^th^ percentile.

##### Households

Food (including dairy) quantities were converted from local units (for example, bunches, liters, packets) to kilograms. Implausible or missing units (<0.67% of observations for any given food) were imputed using the modal value of the commodity-specific units at the smallest aggregation levels (area and region) at which the model could be calculated. Dairy energy, protein, and calcium consumption were calculated using volume/weight conversion factors from the FAO/INFOODS Density Database Version 2.0 [18] and Kenya Food Composition Table 2018 [19]. Household food expenditure was calculated by multiplying the quantity of each food consumed by the food-specific unit value (that is, the reported food price). For foods that did not have a reported food price, we applied the median value per unit of food purchased from broader geographic aggregates (area and region).

We inflated the observed household food and nonfood expenditures to estimate total household expenditure at baseline [20]. Inflation factors (1.24 for food and 1.27 for nonfood items) were calculated as one over the share of the total household food and nonfood budgets that the included items represented for the KHBS households. The calculated total food and nonfood expenditure was winsorized at the 97.5^th^ percentile. All consumption variables were calculated per adult equivalent (AE) to account for differences in household size and composition and winsorized at the 97.5^th^ percentile. AEs were calculated for each survey round by dividing each household member’s age- and sex-specific recommended daily energy intake by the average recommended intake of a 30- to 60-y-old male, 65 kg in weight with a moderate level of physical activity (3,000 kcal/d) [21]. For household food insecurity, we calculated the continuous HFIAS score (range: 0–27) and the categorical indicators [17].

##### Food and milk prices

Food prices were calculated by converting local units of reported household purchases to kilogram weights. Data to convert local units to kilogram weight was compiled from various sources, including market purchases during MoreMilk pre survey planning activities, a recently conducted study in Kenya, and from FAO/INFOODS Density Database Version 2.0 [18,22]. Prices of unpacked milk were obtained from vendor-reported purchase and sales prices over the past 7 d. Extreme values were detected by comparing the distribution of prices to prevailing market prices at the time of the study. Sales prices >100 KSH (or 0.98 USD) per liter (13% of observations) were determined to be infeasible and removed. Extreme values in the reported purchase prices from suppliers were detected in the same way (<1% of observations). No extreme values were detected in follow-up rounds. We expressed all prices in USD, using the exchange rate from November and December 2019 of 102 KSH per USD. The exchange rate was around 106 KSH and 107 KSH per USD at the time of the first and second follow-up surveys, respectively. Price data are presented as mean ± SD and median (IQR).

#### Data *analysis*

##### Attrition analyses

Attrition in longitudinal studies can affect the generalizability of results to the population of interest. In addition, because of high attrition, observed changes in the outcomes by survey rounds may not only reflect the effect of progression of time and the pandemic on those outcomes but also reflect changes in the sample composition across rounds. If participants who opted not to participate in the follow-up rounds had dissimilar (unobserved) outcome values to those that completed the surveys, then the change in sample composition could affect the study conclusions. We used random forest and least absolute shrinkage and selection operator techniques to predict participation in both mobile phone survey rounds based on observable baseline characteristics and to generate weights to adjust outcomes for attrition [[23], [24], [25]].

##### Analysis of changes over time

We fitted longitudinal mixed models with a random intercept by vendor or household for the vendor and household analyses, respectively. The outcomes in the vendor analyses were the volumes of unpacked milk purchased and sold in the last 7 d. The vendor-mixed models included controls for the gender of the business owner, length of business operation at baseline (binary variable with a cut-off at 12 mo), and vendor type (shop/kiosk, milk bar, or street vendor) as covariates. We further explored the odds of dairy business closure (measured as not having sold milk in the 7 d prior to the visit) at each follow-up period using a logistic model that adjusted for the gender of the business owner, vendor type, terciles of baseline milk sales, and length of business operation at baseline (same definitions as above).

The household outcomes were food security, dairy consumption, and the consumption of select other foods. The household-mixed models included controls for terciles constructed from the variable on educational attainment of household head and a centered variable for household AE. To assess whether changes over time differed by household wealth, we conducted the analyses separately by tercile of AE household expenditure.

Food prices were compared across survey rounds to assess the extent to which changing prices might explain changes in household consumption. All analyses were conducted using Stata (Stata version 15.0 software, Stata Corp).

### Ethics

The study was approved by the Institutional Research Ethics Committee of the International Livestock Research Institute (IREC2019-39.1) Nairobi, Kenya, and the institutional review board of the International Food Policy Research Institute (PHND-18-1054M), Washington DC, USA.

## Results

### Effect of attrition on outcome measures

There was considerable attrition between the baseline and the follow-up mobile phone surveys, with 83% (*n* = 200) and 67% (*n* = 161) of the 241 baseline vendors and 67% (*n* = 449) and 50% (*n* = 335) of the 670 baseline households participating in the first and second mobile phone surveys. The main reasons for not participating in the follow-up surveys were that phones were switched off or that respondents did not answer the phone calls. The application of attrition weights did not change our results (Supplemental Figures 1–4), so the analyses presented in this article thus used all available data in each survey round without reweighting.

### Dairy business

At baseline, two-thirds of milk businesses were owned by women (Table 1). Most vendors purchased (rather than produced) the milk to sell, and 4 of 5 had been in the business for >1 y. Only 5% of the participants were street vendors (that is, who sell milk in an open space on the street). Most participants sold milk in shops/kiosks (that is, businesses that sell various products in addition to milk), and few operated a milk bar (that is, businesses only licensed to sell dairy products and eggs). Nearly 3 out of 4 vendors reported having made a profit from their business in the month preceding the baseline survey, with median total sales close to 40 USD/d. Unpacked liquid milk was the most widely sold product, with median sales of ∼20 L/d. In addition to selling unpacked liquid milk, which was a criterion for vendor eligibility in the study, 40 to 50% sold packed yogurt, UHT milk, or pasteurized milk. These products, however, were sold in smaller quantities. The sale price for unpacked milk was around 20% higher than the purchase price.

**TABLE 1:** Vendor baseline characteristics

Vendor characteristics	*n*	% or mean ± SD	Median (IQR)
Female owner	239	67.8	-
Vendor solely owns the business	238	84.0	-
Origin of milk sold			
Own production	239	9.2	-
Purchased milk to sell	239	92.9	-
Type of business			
Shop/kiosk	173	72.4	-
Milk bar	54	22.6	-
Street vendor	12	5.0	-
Business sells other products in addition to dairy	239	74.9	-
Total sales of business yesterday, USD^1^	202	60.3 ± 62.9	40.2 (19.6–78.4)
Business has been operational for <12 mo	238	21	-
Made profit in past month	239	73.6	-
Products sold			
Unpacked milk	239	99.6	-
Unpacked fermented milk	239	29.3	-
Unpacked yogurt	239	2.1	-
Packed pasteurized milk	239	40.6	-
UHT^3^ milk	239	48.5	-
Packed fermented milk	239	20.1	-
Packed yogurt	239	43.5	-
Sale volumes in past 7 d^2^			
Unpacked milk, L	237	242.5 ± 263.8	140.0 (70.0–300.0)
Tercile 1	91	62.2 ± 29.2	68.0 (35.0–90.0)
Tercile 2	67	160.4 ± 33.9	147.0 (140.0–200.0)
Tercile 3	79	520.0 ± 294.7	420.0 (300.0–700.0)
Unpacked fermented milk, L	237	9.5 ± 25.4	0.0 (0.0–5.0)
Unpacked yogurt, L	239	0.0 ± 0.0	0.0 (0.0–0.0)
Packed pasteurized milk, L	237	10.9 ± 20.3	0.0 (0.0–14.0)
UHT milk, L	236	15.5 ± 27.0	0.0 (0.0–21.0)
Packed fermented milk, L	237	1.7 ± 4.5	0.0 (0.0–0.0)
Packed yogurt, L	233	3.9 ± 7.0	0.0 (0.0–5.0)
Prices of unpacked milk in the past 7 d			
Purchase price from farmers, USD/L	119	0.48 ± 0.08	-
Purchase price from dairy coops, USD/L	30	0.50 ± 0.04	-
Purchase price from middlemen, USD/L	72	0.49 ± 0.05	-
Sales price, USD/L	206	0.59 ± 0.06	-

UHT, ultrahigh temperature; USD, United States Dollar.

1 We used a 102 KSH/USD exchange rate (Oct/Nov 2019) to calculate USD values.

2 Volumes for vendors not selling a specific product were set to 0 to calculate the reported means.

3 Ultrahigh temperature processing

Around 15% of vendors had not sold any milk over the 7 d prior to the first and second follow-up surveys, which we interpreted as at least a temporary termination of milk-related business. Vendors selling smaller milk volumes at baseline and those that had been operating the business for a shorter time were more likely to not be selling milk at follow-up (Table 2). The average amount of unpacked milk purchased from suppliers and the amount of milk sold to customers by vendors who continued selling milk during the follow-up period dropped by around 30% (*P* < 0.05) at the first follow-up (Supplemental Table 1; Table 3). The point estimates for the unpacked milk volumes supplied and sold at second follow-up were ∼15% lower than at baseline (NS, for purchases; *P* <0.05 for sales). When including vendors who stopped selling milk in the analyses (setting their unpacked milk volumes to 0), the changes over time were even more pronounced. The findings were similar across the 3 types of vendors in the study, although the small sample size prevented any meaningful statistical subgroup analysis (Table 3). Self-reported reasons for vendors’ difficulties sourcing milk differed across the 2 follow-up periods. In the first follow-up survey, one-third of the study vendors who reported having difficulties sourcing milk attributed it specifically to challenges with transportation and business operations restrictions along with lower milk availability. By the second round of interviews, most of the vendors who reported difficulty in sourcing milk no longer listed transportation challenges and business restrictions as primary reasons for their supply shortages (Supplemental Table 3) and indicated instead a decrease in milk availability.

**TABLE 2:** Odds of businesses not selling milk in the past 7 d and prevalence difference by baseline vendor characteristics at each follow-up survey

Variables	Follow-up 1	Follow-up 2
	*n* = 196	*n* = 156
Female owner	1.35 (0.70)	0.73 (0.44)
Type of business		
Shop/kiosk	ref.	ref.
Milk bar	1.92 (1.26)	1.72 (1.89)
Street vendor	1.09 (0.96)	1.33 (1.22)
Sale volumes of unpacked milk in past 7 d		
Tercile 1	ref.	ref.
Tercile 2	0.67 (0.34)	0.37 (0.23)
Tercile 3	0.30 (0.20)∗	0.10 (0.11)∗∗
Business in operation for <12 mo	2.83 (1.32)∗∗	2.74 (1.58)∗
Constant	0.14 (0.07)∗∗∗	0.28 (0.16)∗∗
X^2^ test for likelihood ratio	11.52∗	11.97∗

Values are odds ratios (SE); ∗∗∗*P* < 0.01, ∗∗*P* < 0.05, ∗*P* < 0.1. ref., reference.

**TABLE 3:** Vendor changes in volume of milk supplied and sold over time

Milk volumes	Wave	All vendors	Stratified by vendor type		
			Shop/kiosk	Milk bar	Street vendor
Total volume - vendors still in business		*n* = 540	*n* = 394	*n* = 116	*n* = 30
Unpacked milk purchases, past 7 d, L	1	154.9 (117.9–192.0)	149.6 (118.6–180.6)	505.0 (368.9–641.2)	214.8 (59.9–369.7)
	2	109.9 (70.9–148.8)∗∗	131.2 (98.6–163.7)	376.7 (232.7–520.8)∗∗	153.9 (−10.1 to 317.8)
	3	139.4 (99.0–179.8)	152.6 (119.0–186.2)	453.1 (300.7–605.5)	104.9 (−69.2 to 279.0)
		*n* = 516	*n* = 376	*n* = 113	*n* = 27
Unpacked milk sales, past 7 d, L	1	157.9 (120.9–195.0)	160.2 (128.2–192.3)	465.3 (333.4–597.3)	227.7 (71.5–384.0)
	2	108.3 (68.6–147.9)∗∗	119.0 (84.9–153.0)∗∗	396.5 (257.0–536.0)	195.4 (−2.7 to 393.6)
	3	134.7 (95.1–174.4)	143.2 (109.3–177.1)	449.9 (305.1–594.6)	124.6 (−55.5 to 304.8)
Total volume - all vendors		*n* = 591	*n* = 432	*n* = 125	*n* = 34
Unpacked milk purchases past 7 d, L	1	157.5 (120.5–194.6)	148.6 (118.2–178.9)	499.6 (362.3–636.9)	232.7 (66.1–399.4)
	2	92.4 (54.5–130.3)∗∗	115.9 (84.9–146.9)∗∗	333.4 (191.9–474.9)∗∗	129.0 (−43.9 to 301.8)
	3	124.3 (85.1–163.6)∗	135.3 (103.2–167.4)	423.6 (273.5–573.8)	103.1 (−69.8 to 275.9)∗
		*n* = 568	*n* = 415	*n* = 122	*n* = 31
Unpacked milk sales, past 7 d, L	1	158.4 (121.4–195.3)	157.1 (125.8–188.3)	460.6 (326.5–594.6)	241.5 (69.2–413.9)
	2	88.7 (50.2–127.2)∗∗	104.2 (71.7–136.7)∗∗	346.5 (208.2–484.9)∗∗	127.8 (−69.5 to 325.2)
	3	119.7 (81.0–158.4)∗∗	126.8 (94.2–159.3)∗∗	420.6 (276.1–565.1)	105.1 (−74.2 to 284.3)∗

Values are mean (95% CI). *N* is the number of observations in each model. Statistical tests compare waves 2 and 3 to baseline (wave 1), where ∗*P* < 0.05; ∗∗*P* < 0.01.

### Household dairy and food consumption

Households were small, and most were headed by a male (Table 4). Most household heads had completed primary education and worked for pay. Mean per AE daily total household expenditure varied from USD 2.22 in the lowest expenditure tercile to USD 5.65 in the highest tercile.

**TABLE 4:** Household baseline characteristics

Household characteristics	*n*	% or mean ± SD
Size	670	4.4 ± 1.4
Head of household age, y	663	34.4 ± 8.5
Head of household, % male	664	79.7
Head of household education		
Primary or less	199	30.0
Some or complete secondary	324	48.8
Higher education	141	21.2
Head of household main activity past 7 d		
Worked for own/family business	166	25.0
Worked for pay	432	65.1
Homemaker	27	4.1
Other	39	5.9
Daily total per AE household expenditure–all households, USD	670	3.82 ± 1.60
Daily total per AE household expenditure–tercile 1, USD^1^	224	2.22 ± 0.54
Daily total per AE household expenditure–tercile 2, USD^1^	223	3.61 ± 0.39
Daily total per AE household expenditure–tercile 3, USD^1^	223	5.65 ± 1.13
Prevalence of food insecurity, %	670	70.0

AE, adult equivalent; USD, United States Dollar.

1 Terciles were created on the basis of total per AE household expenditure.

At baseline, 7 of 10 households were food insecure.

The most consumed dairy product was unpacked milk: households reported consuming an average of 330 mL of unpacked milk per AE per day (Supplemental Table 2). All dairy products combined provided around 10% of the recommended daily energy intake of the reference adult and a total of 13 g of protein per day. Around 40% of households reported having purchased foods away from home, and 25% purchased soft drinks in the week preceding the survey.

**FIGURE 1:**
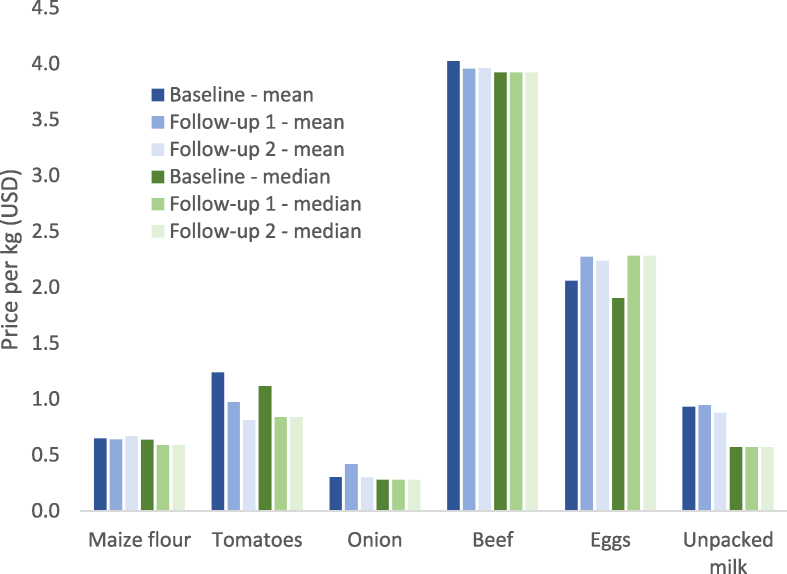
Mean and median food prices over time. USD, United States Dollar.

Relative to the baseline, the prevalence of food insecurity increased by 16 and 11 percentage points in the first and second follow-up surveys, respectively (*P* < 0.01) (Table 5). Maize flour consumption increased by 8% in the first follow-up (*P* < 0.01) and returned to baseline levels by the second follow-up. Tomato consumption remained stable over time, but tomato-related expenditures declined. Onion consumption declined and only partially recovered by the second follow-up survey. Relative to baseline, households had decreased their daily consumption of beef by 22% (*P* < 0.01), eggs by 15% (*P* < 0.05), and unpacked milk by 23% (*P* < 0.01) at the first follow-up. At the second follow-up, consumption of these animal source products was still significantly lower than that during the baseline survey conducted a year earlier. The reported reduction in dairy consumption implies a decrease in protein and calcium consumption from this food group of around 3 g and 110 mg per AE, respectively. The proportion of households consuming foods away from home and soft drinks declined significantly at the time of the first follow-up survey and remained slightly lower than baseline at the time of the second follow-up. Food prices for maize flour, onions, beef, and unpacked milk remained stable over time (Figure 1). The median price of tomatoes dropped by 25%, and the price of eggs increased by 20%.

**TABLE 5:** Household outcomes: adjusted changes over time

Variables	Wave	All households	Stratified by terciles of household expenditure		
			Lower tercile	Middle tercile	Upper tercile
*n*		1445	500	486	459
Food insecurity					
Prevalence of food insecurity, %	1	71.4 (66.4–76.5)	86.2 (80.6–91.7)	63.9 (54.2–73.6)	50.4 (38.1–62.7)
	2	87.7 (82.2–93.1)∗∗	93.8 (87.7–99.9)∗∗	82.1 (71.7–92.4)∗∗	73.3 (60.2–86.4)∗∗
	3	82.6 (76.7–88.5)∗∗	85.1 (78.4–91.8)	83.8 (72.7–94.9)∗∗	65.7 (52.1–79.3)∗∗
Staple consumption					
Monthly per AE maize flour consumption, g	1	5558.4 (5201.0–5915.7)	5274.3 (4722.3–5826.3)	5409.0 (4741.7–6076.3)	5994.4 (5273.2–6715.5)
	2	6018.0 (5633.6–6402.4)∗∗	5835.3 (5234.3–6436.3)∗	5914.2 (5203.8–6624.6)	6302.5 (5535.4–7069.6)
	3	5641.0 (5230.9–6051.1)	5506.8 (4854.7–6158.9)	5348.8 (4592.6–6105.0)	6026.9 (5229.5–6824.2)
Monthly per AE maize flour consumption, USD	1	3.63 (3.39–3.87)	3.46 (3.07–3.86)	3.55 (3.11–3.98)	3.71 (3.23–4.18)
	2	3.82 (3.56–4.08)	3.90 (3.47–4.33)∗	3.69 (3.23–4.15)	3.70 (3.19–4.20)
	3	3.49 (3.22–3.77)	3.58 (3.11–4.04)	3.32 (2.82–3.81)	3.37 (2.85–3.90)
Vegetables					
Monthly per AE tomato consumption, g	1	1981.9 (1856.3–2107.5)	1496.4 (1337.4–1655.5)	2171.6 (1946.4–2396.8)	2463.9 (2190.9–2737.0)
	2	1901.5 (1766.4–2036.7)	1680.3 (1506.8–1853.7)∗	2030.1 (1790.4–2269.8)	2184.4 (1892.6–2476.2)∗∗
	3	1969.5 (1825.3–2113.6)	1720.3 (1531.8–1908.9)∗∗	2223.4 (1968.2–2478.5)	2128.4 (1824.2–2432.6)∗∗
Monthly per AE tomato consumption, USD	1	2.28 (2.14–2.42)	1.63 (1.47–1.78)	2.45 (2.20–2.71)	3.12 (2.80–3.45)
	2	1.65 (1.50–1.81)∗∗	1.42 (1.25–1.59)∗∗	1.69 (1.41–1.97)∗∗	2.22 (1.87–2.56)∗∗
	3	1.51 (1.34–1.67)∗∗	1.35 (1.16–1.54)∗∗	1.66 (1.37–1.96)∗∗	1.83 (1.47–2.19)∗∗
Monthly per AE onion consumption, g	1	3225.5 (3044.6–3406.4)	2642.4 (2411.2–2873.5)	3530.1 (3206.6–3853.6)	3735.4 (3342.6–4128.2)
	2	2712.0 (2515.3–2908.6)∗∗	2350.4 (2094.8–2606.0)∗	3103.5 (2755.2–3451.7)∗∗	2902.9 (2479.4–3326.4)∗∗
	3	3012.7 (2800.8–3224.7)∗	2541.1 (2259.1–2823.2)	3351.5 (2976.1–3726.9)	3351.3 (2907.7–3794.9)∗
Monthly per AE onion consumption, USD	1	0.93 (0.86–0.99)	0.73 (0.65–0.80)	1.05 (0.93–1.17)	1.13 (0.99–1.28)
	2	0.94 (0.87–1.01)	0.76 (0.67–0.84)	1.09 (0.96–1.22)	1.12 (0.97–1.28)
	3	0.84 (0.76–0.92)∗	0.68 (0.59–0.77)	0.99 (0.85–1.13)	0.99 (0.83–1.15)∗
Animal source foods					
Monthly per AE beef consumption, g	1	795.3 (688.3–902.2)	396.2 (289.0–503.5)	868.0 (672.0–1064.0)	1414.3 (1160.0–1668.7)
	2	627.3 (512.4–742.2)∗∗	448.7 (330.9–566.4)	723.1 (514.1–932.1)	1003.1 (732.7–1273.4)∗∗
	3	623.4 (500.9–745.8)∗∗	445.3 (316.4–574.2)	796.2 (573.3–1019.2)	896.3 (615.4–1177.1)∗∗
Monthly per AE beef consumption, USD	1	3.13 (2.72–3.55)	1.57 (1.17–1.97)	3.63 (2.87–4.38)	5.30 (4.29–6.30)
	2	2.45 (2.00–2.90)∗∗	1.72 (1.28–2.16)	3.02 (2.21–3.82)∗	3.72 (2.65–4.79)∗∗
	3	2.54 (2.07–3.02)∗∗	1.70 (1.23–2.18)	3.33 (2.47–4.19)	3.63 (2.52–4.74)∗∗
Monthly per AE egg consumption, g	1	338.4 (290.0–386.7)	190.7 (147.5–233.9)	408.8 (323.7–494.0)	502.1 (373.5–630.7)
	2	286.6 (234.6–338.7)∗	223.9 (176.2–271.6)	371.8 (280.7–463.0)	346.4 (210.0–482.9)∗∗
	3	291.1 (235.5–346.7)∗	207.3 (154.7–259.8)	380.7 (283.1–478.4)	367.9 (226.3–509.5)∗∗
Monthly per AE egg consumption, USD	1	0.69 (0.59–0.80)	0.38 (0.29–0.47)	0.84 (0.66–1.03)	1.05 (0.78–1.33)
	2	0.62 (0.51–0.74)	0.48 (0.38–0.58)	0.83 (0.63–1.03)	0.75 (0.46–1.05)∗∗
	3	0.66 (0.54–0.78)	0.44 (0.33–0.55)	0.85 (0.64–1.06)	0.87 (0.57–1.17)
Dairy					
Daily per AE unpacked milk consumption, ml	1	326.8 (306.7–346.9)	264.3 (240.4–288.2)	342.6 (307.7–377.4)	405.7 (357.0–454.5)
	2	251.1 (229.5–272.7)∗∗	226.4 (200.2–252.7)∗∗	256.3 (219.1–293.4)∗∗	303.5 (251.7–355.2)∗∗
	3	241.1 (218.0–264.2)∗∗	222.0 (193.2–250.8)∗∗	251.1 (211.4–290.8)∗∗	279.1 (225.3–332.8)∗∗
Monthly per AE unpacked milk consumption, USD	1	5.86 (5.51–6.21)	4.72 (4.32–5.13)	6.24 (5.63–6.85)	7.19 (6.34–8.03)
	2	4.59 (4.21–4.96)∗∗	4.10 (3.65–4.54)∗∗	4.85 (4.20–5.50)∗∗	5.40 (4.51–6.30)∗∗
	3	4.45 (4.05–4.85)∗∗	4.18 (3.70–4.67)∗	4.70 (4.00–5.40)∗∗	4.99 (4.06–5.92)∗∗
Monthly per AE dairy consumption, USD	1	7.69 (7.23–8.16)	5.89 (5.38–6.41)	7.99 (7.21–8.77)	10.35 (9.29–11.42)
	2	5.83 (5.33–6.33)∗∗	4.95 (4.38–5.51)∗∗	6.17 (5.34–7.00)∗∗	7.54 (6.41–8.67)∗∗
	3	5.74 (5.21–6.27)∗∗	4.90 (4.28–5.52)∗∗	6.04 (5.15–6.93)∗∗	7.37 (6.19–8.54)∗∗
Daily per AE energy from dairy, kcal	1	275.4 (259.0–291.8)	218.0 (199.2–236.8)	285.4 (257.2–313.6)	360.2 (322.4–397.9)
	2	212.0 (194.5–229.6)∗∗	182.8 (162.2–203.5)∗∗	215.2 (185.2–245.3)∗∗	276.9 (236.9–317.0)∗∗
	3	204.4 (185.7–223.0)∗∗	175.6 (153.0–198.2)∗∗	211.4 (179.4–243.5)∗∗	261.3 (219.8–302.8)∗∗
Daily per AE protein from dairy, g	1	12.7 (12.0–13.5)	10.2 (9.3–11.0)	13.2 (11.9–14.5)	16.4 (14.7–18.2)
	2	9.9 (9.1–10.7)∗∗	8.5 (7.6–9.5)∗∗	10.0 (8.6–11.4)∗∗	12.7 (10.9–14.6)∗∗
	3	9.5 (8.7–10.4)∗∗	8.2 (7.2–9.3)∗∗	9.8 (8.4–11.3)∗∗	12.1 (10.2–14.0)∗∗
Daily per AE calcium from dairy, mg	1	464.7 (437.0–492.3)	369.0 (337.1–401.0)	482.5 (434.8–530.2)	603.8 (540.0–667.6)
	2	360.5 (330.9–390.2)∗∗	309.6 (274.6–344.7)∗∗	364.7 (313.9–415.6)∗∗	471.5 (403.9–539.0)∗∗
	3	348.3 (316.8–379.8)∗∗	297.5 (259.1–335.9)∗∗	359.9 (305.7–414.1)∗∗	446.0 (376.0–516.0)∗∗
Other food					
Expended/consumed food away from home in the past week, %	1	39.4 (34.0–44.9)	31.3 (23.4–39.2)	42.4 (32.0–52.8)	51.7 (40.2–63.2)
	2	22.3 (16.4–28.3)∗∗	25.6 (16.9–34.3)	24.4 (13.2–35.5)∗∗	24.3 (11.9–36.7)∗∗
	3	30.1 (23.7–36.5)∗∗	25.4 (15.8–35.0)	36.3 (24.3–48.2)	36.5 (23.5–49.5)∗∗
Expended/consumed soda in the past week, %	1	21.7 (16.9–26.5)	14.4 (8.9–20.0)	18.0 (9.1–27.0)	40.2 (28.1–52.4)
	2	10.7 (5.4–15.9)∗∗	8.6 (2.4–14.7)	8.0 (−1.6 to 17.6)∗∗	22.4 (9.6–35.3)∗∗
	3	14.0 (8.3–19.6)∗∗	10.7 (3.8–17.5)	14.8 (4.5–25.1)	22.3 (8.9–35.6)∗∗

Values are mean (95% CI). *N* is the number of observations in each model. Statistical tests compare waves 2 and 3 to baseline (wave 1), where ∗*P* < 0.05; ∗∗*P* < 0.01. AE, adult equivalent; USD, United States Dollar.

Steep increases in the percentage of food insecure households were seen across all 3 expenditure-based terciles at the time of the first follow-up survey, with an additional 7%, 20%, and 23% of households in the lower, middle, and upper terciles being food insecure, respectively (Table 5). By the time of the second follow-up survey, the prevalence of food insecurity had returned to prepandemic levels in the lowest tercile, but the prevalence of food insecurity remained significantly higher than that at baseline in the other 2 expenditure terciles (*P* < 0.01). Changes over time in consumption were seen across all expenditure groups but were larger in both absolute and relative terms in the highest expenditure tercile.

## Discussion

Using data collected before and after the onset of the COVID-19 pandemic, we found important changes in milk sales in the informal dairy sector and in the consumption of dairy products by low-income households. About 15% of dairy vendors operating in the informal market had (temporarily or permanently) closed their dairy-related business 4 mo after the onset of the pandemic. A similar percentage reported closure 1 y after baseline. Closure was more likely among vendors who sold lower amounts of milk and those who had been in the business for <1 y at baseline. At baseline, 21% of our study vendors reported having been in business for fewer than 12 mo. Similar rates have been found in previous studies in the area [26]. These observed rates of closure are consistent with the high turnover among informal businesses in the dairy sector, with newer and smaller businesses being the most vulnerable. This frequent turnover of small informal dairy businesses is driven, in part, by the marked seasonality of milk production: new businesses open and operate for only few months when milk availability is high, easy to source, and relatively cheap. Moreover, the high demand for milk in Kenya and the relatively low business start-up costs make it an attractive venture that can provide easy money with little investment [26]. Because closure rates observed in our study were similar to those in prepandemic times, our study provides no evidence that COVID-19 contributed to increased business closures.

**FIGURE 2:**
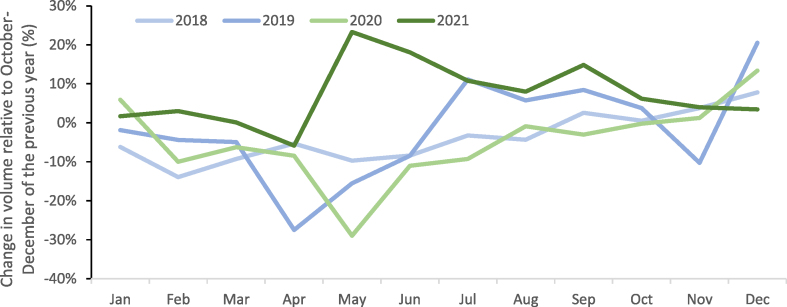
Variation in the monthly volume of milk received by processors in Kenya [28].

We found a sharp decrease in the volume of liquid milk sold by dairy vendors in the study. Whereas this decrease was most pronounced in the first follow-up survey—roughly 4 mo after the onset of the pandemic—sales had not completely recovered 9 mo into the pandemic. Milk production is typically highest from November to January, in response to the major rainy season that happens from October to December, declines to its lowest levels between April and May, and then increases gradually for the rest of the year, following the short rainy season of March and April [27]. This seasonal change in milk availability is also observed in the Kenya Dairy board’s monthly records of the amount of milk received by processors, a proxy for milk production [28]. In the 2 y prior to the pandemic, milk volumes in July were 3% lower to 11% higher compared with the months of October, November, and December of the preceding year (Figure 2). In July 2020, the volume of milk received by processors was 9% lower. It is therefore unlikely that seasonal variations in milk availability accounted for the observed 30% decrease in milk sales by informal vendors at the time of the first follow-up in July 2020. Likewise, seasonality is unlikely to explain the 15% reduction in milk sales by the informal vendors in the study at the second follow-up: milk availability (as measured by the volume of milk received by processors) in September 2020–October 2020 was approximately the same as at the time of the baseline survey (October 2019–December 2019). Government restrictions and individual behavioral responses to the COVID-19 pandemic may therefore have contributed to the decrease in milk value chain activity. Several studies, mostly conducted among dairy businesses operating in the formal sector, such as cooperatives and industrial processors, reported that formal restrictions in Kenya did not substantially affect the flow of milk in those formal dairy supply chains beyond the first 2 wk of restrictions [29,30]. Qualitative evidence from our study, however, indicates that informal markets may have been more affected by these restrictions. Vendors attributed their challenges sourcing milk to transportation and business restrictions at the first follow-up (when restrictions were more severe) and did not identify these restrictions as impediments to milk sourcing during the second follow-up (when the restrictions had been relaxed). The larger effect of COVID-19–related restrictions on the informal (as compared to the formal) dairy sector was also documented by others [30].

The reduction in the amount of milk sold by informal vendors is consistent with our finding that household consumption of unpacked milk declined significantly. Our study does not allow us to establish if the reduction of milk sold in the markets was driven by a decrease in household demand or if the reduction of milk consumption by households was a response to a decrease in milk supply. A limitation of our study is that the reduction in the volume of milk sold by vendors cannot be interpreted as a reflection of the true reduction in milk availability in the market in the study areas because new market entrants are also likely to be contribute to total market supply. Whereas our study showed that some businesses closed throughout the study period and those open were selling lower amounts than expected, we do not know to what extent new milk outlets may have appeared in the study area. The drop in household milk consumption, on the other hand, is not subject to the same concerns and offers some support for the idea that our observed decrease in milk supply may not be driven by our incomplete enumeration of existing milk vendors at the time of the 2 follow-up surveys.

Our household data showed a steep increase in the prevalence of food insecurity and a large reduction in the consumption of animal source foods between baseline and follow-up surveys. The changes in these outcomes observed at the first follow-up (when strict COVID-19 measures were still in place) were similar to those observed at the second follow-up when most restrictions had been lifted. Consumption of maize flour increased and subsequently returned to prepandemic levels. Patterns for the 2 vegetables we asked about differed: tomato consumption stayed stable whereas onion consumption dropped. Although we did not assess household income directly, our data suggest that these changes in household consumption may have been accompanied by a loss in earnings. When asked about the largest impact of the COVID-19 crisis, most households identified loss of income (80% and 88% of households in the first and second follow-up survey, respectively; results not shown).

The largest absolute and relative changes in food insecurity and animal source food consumption were observed in the wealthiest households in the study sample. By the time of the second follow-up survey, the prevalence of food insecurity had returned to prepandemic levels among the poorest households but remained significantly higher than at baseline in the wealthiest ones. Similar patterns were observed for the consumption of animal source foods: in the poorest households, the consumption of beef and eggs remained at stable (low) levels over time; in the highest expenditure tercile, consumption per AE per day dropped by 14 g and 17 g for beef and by 5 g and 4 g for eggs at the first and second follow-ups, respectively. A decrease in unpacked milk consumption was seen across expenditure levels, but it was considerably larger in the highest expenditure tercile (a drop of >100 mL per AE per day) than in the poorest households (around 40 mL per AE per day). At the time of the second follow-up, unpacked milk consumption in the wealthiest households was comparable to the prepandemic consumption levels in the middle expenditure tercile. Likewise, consumption in the middle tercile group had dropped to the prepandemic levels in the poorest households. Even though per AE consumption of animal source foods remained highest in the wealthiest households, the difference in consumption relative to the poorest households became smaller over time. Whereas we do not have a clear explanation for why the effects were more pronounced for (relatively) wealthier households, it is worth emphasizing that there may have been less scope for reductions in animal–sourced food expenditure for the poorest households as they were already spending a tiny fraction of the amount spent by the wealthier sample households.

The household consumption data for selected foods do not allow us to accurately quantify the effect of these changes on household nutrient intake. The decrease in the consumption of milk and beef, however, is of concern. These animal source foods are excellent sources of protein, vitamin A, vitamin B-12, riboflavin, zinc, and calcium [31]. Daily per AE consumption of protein from unpacked milk in our sample, for instance, dropped by around 2, 3, and 4 g in the bottom, middle, and top tercile, respectively. Given the consistent decrease observed for milk, beef, and egg consumption, it is unlikely that households increased the consumption of other types of animal source foods (such as chicken or pork). Likewise, households did not shift from unpacked milk to other dairy products (data not shown). Consequently, we believe that the quality of household diets is likely to have declined over the study period (that is, from the end of 2019 to the end of 2020). The true decline in dietary quality could even be larger than what we observed. Because households consumed more meals at home due to government-imposed restrictions, a larger proportion of total household food consumption was likely captured by our food expenditure module at the time of follow-ups. Our comparison with baseline observations may thus underestimate the actual changes over time.

Two of the observed changes could have positive implications from a nutrition perspective. First, fewer households reported eating food and meals away from home. Street foods are often high in fat, sugar, and salt [32]. Second, the proportion of the study households consuming sugar-sweetened beverages decreased. The consumption of these drinks is associated with risk of weight gain, diabetes, and metabolic conditions [33]. These noncommunicable diseases impose a large and growing burden on societies in Africa South of the Sahara [34].

A key strength of our study is that baseline data were collected (shortly) before the onset of the pandemic and that we do not rely on respondent recall about the pre–COVID-19 situation. The change in data collection method (from in-person to phone surveys) is an inherent limitation of our study. We intentionally kept phone surveys to a maximum of 20 min to reduce respondent fatigue. We have no reason to believe that the change in methods biased the results in any particular direction. Because our findings are mixed (for example, we find changes in the household consumption of only some of the foods included in the survey) and are consistent across the household and vendor samples, we are confident that our findings are not simply the result of respondent bias.

Several high-level publications have highlighted the association between the COVID-19 pandemic and economic hardship for households in low- and middle-income countries [35]. Rigorous evidence based on data collected (shortly) before and after the onset of the pandemic, however, is limited. In Ethiopia, an in-person survey conducted before the onset of the pandemic was followed by several phone surveys. Most households in the study panel reported income loss; however, the authors found no clear evidence of a change in the quality of the household diet. Five months after the start of the pandemic, the consumption of staples had risen whereas the consumption of legumes and vegetables had fallen. Importantly, fruit and animal source food consumption remained the same on average [36]. It is not clear to what extent, reductions in the consumption of foods away from home may mask drops in the quality or quantity of household food consumption. In rural households in Guatemala’s highlands, a steep increase in food insecurity and a decrease in household dietary diversity was found 2–3 mo after the start of the pandemic. The study used data from in-person pre and phone postsurveys. Households consumed fewer animal source foods but increased the diversity of fruit and vegetable consumption. Similar to our findings, relatively wealthier households showed the largest reductions in dietary diversity [37]. Finally, a study in rural Kenya used household expenditure diaries collected from February to April 2020, that is, in the 5 wk before and 5 wk immediately after the outbreak started. Income from work declined, but no change in household food expenditure was observed. Households may have been able to maintain food spending in the short term by reducing nonfood expenditures and by relying on savings or remittances. This type of response, however, may have become more difficult as the crisis continued [38].

Our observational before and after study design does not allow us to infer causality, so we do not know to what extent the changes in volumes of milk sold in the market and milk consumption by households we observed were due to the COVID-19 pandemic or associated restrictions on behavior. However, we argue that it is plausible that part of the observed change is attributable to the COVID-19 pandemic. First, the findings are unlikely to be driven entirely by seasonality (Figure 2). The baseline and second follow-up were conducted at approximately the same time of year, but important differences in outcomes were nevertheless observed among vendors and households. We cannot exclude the possibility that confounding factors drive our findings, but we are not aware of policies or programs implemented in the study area that would have led to the changes observed in our data and cannot think of any confounders that would explain the observed patterns. An increase in the price of animal source foods unrelated to COVID-19, for example, would have resulted in a reduction in consumption, but food prices were remarkably stable over time. Stable food prices during the study period in Kenya have also been reported elsewhere [39]. Finally, the deterioration of household food security in Kenya following the start of the pandemic is consistent with other evidence [39].

What are the implications of our work under the assumption that the observed changes were indeed due to the COVID-19 pandemic? In times of crisis, short-term relief efforts and longer-term social protection policies should be implemented to maintain low-income household purchasing power and consequently protect the quality of the diet, which appeared highly sensitive to the shock induced by the pandemic. These efforts should not just target the poorest of the poor. Nearly 90% of the households in our study lived above the international poverty line (USD 1.90 per person per day) at baseline. In addition, even though the wealthiest households in the sample at baseline remained better off in terms of household food security and food consumption throughout the study period, the largest absolute and relative changes were seen in this group. By helping households maintain the quality of their diets, these policies should also be expected to have positive effects on food vendors and other value chain actors, as the demand for highly nutritious products would be maintained. Finally, movement restrictions should be designed to minimize possible disruptions to food supply chains, in particular of fresh foods, which are more vulnerable to supply chain disruptions. This will both ensure an adequate supply of nutritious products in the market but also protect the incomes of those individuals who depend on informal markets for their livelihood.

## Funding

This publication is based on research funded by the Bill & Melinda Gates Foundation and UK aid from the UK government (Ref. OPP1156625). The findings and conclusions are those of the authors and do not necessarily reflect the positions or policies of the Bill & Melinda Gates Foundation or the UK government. The study also received financial support from the CGIAR Research Program on Agriculture for Nutrition and Health. The funders had no role in the design of the study, collection, analysis, and interpretation of data or in the writing of this publication. There were no restrictions on the publication of this report.

## Author disclosures

The authors report no conflicts of interest.

## Data Availability

Data described in the manuscript, code book, and analytic code will be made available upon request.
