# Supplementary material for: Consumer Demand for Milk and the Informal Dairy Sector Amidst COVID-19 in Nairobi, Kenya
Source: Curr Dev Nutr. 2023 Feb 25;7(4):100058. doi: 10.1016/j.cdnut.2023.100058 (PMC9957657; doi:10.1016/j.cdnut.2023.100058)

**Attrition analyses**

There was considerable attrition between the baseline and the follow-up mobile phone surveys with 67% of vendors and 50% of households participating in the final mobile phone survey. As a result, comparing the evolution of outcomes over time using all available data will show both changes due to the progression of time, COVID-19 pandemic, and the imposition of restrictions and changes due to differences in the sample composition across rounds. If households that opt not to participate in the follow-up rounds have dissimilar—and, to us, unobserved—outcome values, then the change in sample composition will affect the study conclusions. If, however, sample attrition is unrelated to the outcome values, then using all collected data in each round will not change the study take-aways and will yield more precise estimates. Because we do not observe values for households that attrit from the sample, direct assessments of whether there are differences in outcomes in the mobile phone survey rounds are not feasible. Instead, we rely on indirect assessments of likely imbalance and explore the robustness of our estimates to methods that aim to correct for non-random sample attrition.

A common approach to remove the potential bias resulting from attrition is to reweight observations that are observed across all survey rounds based on their estimated probability of participating in all survey waves. This method gives more weight to observations that look similar at baseline to households that were more likely to attrit and corrects for potential bias if attrition is as good as random conditional on the characteristics used in the model. Attrition is typically modeled by estimating a binary dependent variable model (e.g. probit or logit) with controls selected based on researcher priors. The model-based predicted probabilities of participation are then inverted, normalized, and used as weights (known as inverse probability weighting).

Recent developments in statistical learning offer useful alternatives for adjusting for possibly non-random attrition; in particular, supervised learning techniques can provide more accurate predictions for a given dataset and research question than the less flexible methods more commonplace in the literature (39–41). We used a random forest (RF) machine learning algorithm—an average of many intentionally de-correlated classification and regression trees (CART)—to predict participation in both mobile phone survey rounds and generate weights to adjust outcomes for attrition. We also show estimates from a logit with controls selected via least absolute shrinkage and selection operator (LASSO) and results from a propensity to attrit analysis based purely on our priors.

*Selecting variables for RF and LASSO*

Selection of predictor variables from the baseline survey was based on the expectation that they might predict attrition and on having sufficient sample variation. For dairy vendors, we included variables on business characteristics (e.g. type of dairy vendor, gender of business owner, licensed to sell, ownership and quality of business site, business services, co-ownership of business, and time in business), business capital and revenue, milk sourcing and handling practices, and dairy sales. For households, we included variables on household composition and demographics, housing quality and services, household dietary diversity and food insecurity, dairy expenditures, household food and non-food expenditures, mother’s knowledge on nutrition and health, child health and morbidity, and hygiene and sanitation practices.

We replaced missing values with the sample median and replaced categorical variables with a re-ordered variable based on the mean probability of full participation in each category. For example, a variable with original categories 0, 1, and 2 was re-ordered so that the group with value 2 had the highest participation rate, the group with value 1 had the second highest participation rate, and the group with value 0 had the lowest participation rate. The reordered variable was treated as continuous. In addition to the set of variables described here, the LASSO models included the square of each variable and the interaction between each variable and all other variables. In total, we included 74 and 2,820 variables in the RF and LASSO models to predict household attrition, respectively, and we included 53 and 1,458 variables in the RF and LASSO models to prediction vendor attrition, respectively.

*Predicting attrition from baseline household characteristics*

We implemented RF regression using ‘crtrees’ Stata command, and generated average predictions of follow-up participation using baseline predictors. The total number of trees grown was set to 500, and the number of variables at each node was set to the square root of the number of predictors.

We implemented first-stage LASSO using ‘lasso’ Stata command with a 10-fold cross-validation to select the penalty term λ. We then predicted probability of participation in each survey round and in all survey rounds.

*Application of inverse probability weights*

Inverse probability weights were calculated as 1/the predicted probability of participation in the mobile phone survey(s). These raw weights were normalized by multiplying each weight by (sample size/sum of the raw weights).

We considered two approaches to reweighting the data. The first approach uses all the data we have in each survey round and re-weights the samples in round 2 and round 3 using weights calculated separately for each survey round. The second approach uses weights calculated from baseline predictors of the full sample based on the likelihood that households participated in all survey rounds, and then shows how outcomes change over time for this balanced panel (after re-weighting). For comparison, we present unweighted results of the balanced panel.

We applied the weights to two main outcomes of the vendor and household surveys. These are quantities (liters) of unpacked milk that vendors sourced from milk suppliers in the past 7 days, quantities (liters) of unpacked milk vendors sold in the past 7 days, household food insecurity and access scores (HFIAS), and household dairy expenditures per adult equivalent.

*Results*

Figures s1 and s2 show mean quantities (liters) of unpacked milk sourced from suppliers and sold to customers from unweighted and weighted analyses. In all cases, results are insensitive to weights from baseline predictors of attrition, from method of weights produced (lassos or random forests) and to whether or not results are restricted to vendors participating in all survey rounds (balanced panel).

Figures s3 and s4 show results from household dairy expenditures per adult equivalent and mean HFIAS score for both unweighted and weighted analyses. Mean dairy expenditures per adult equivalent and mean HFIAS scores are not affected by weights generated from either lasso or random forest produced weights, nor are they meaningfully affected by whether or not results are restricted solely to the households participating in all survey rounds.

**Figures**

**Figure s1: Quantity (liters) of unpacked milk vendor sourced from suppliers in past 7 days**


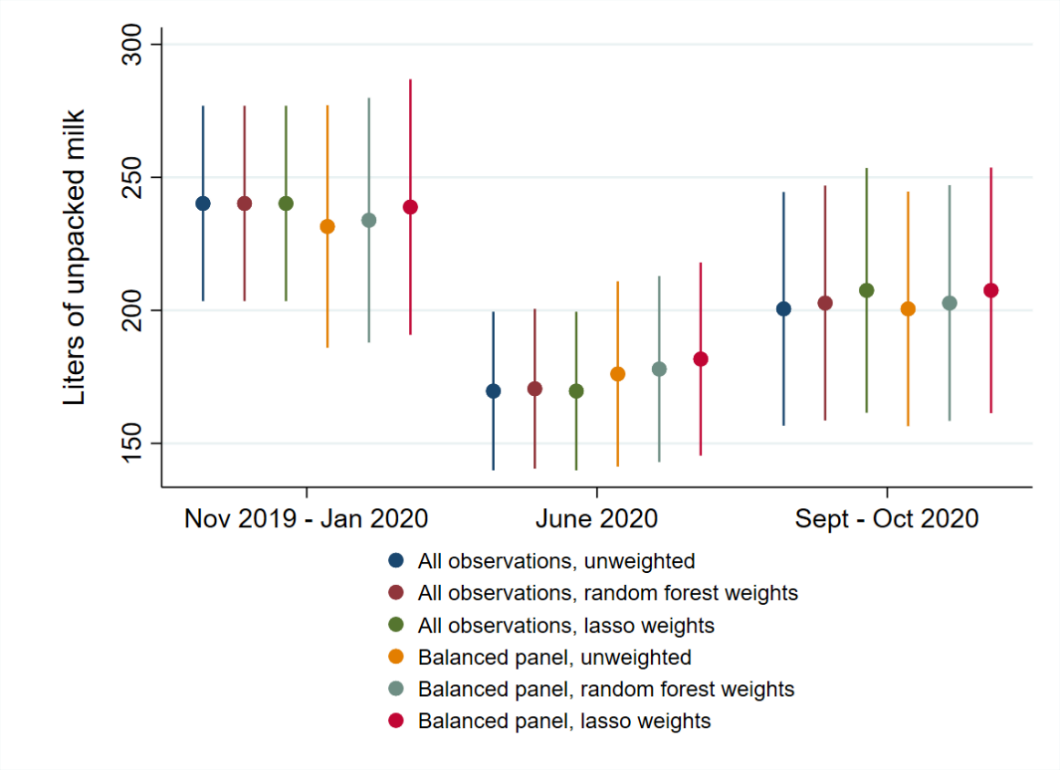


**Figure s2: Quantity (liters) of unpacked milk from vendor sold in past 7 days**


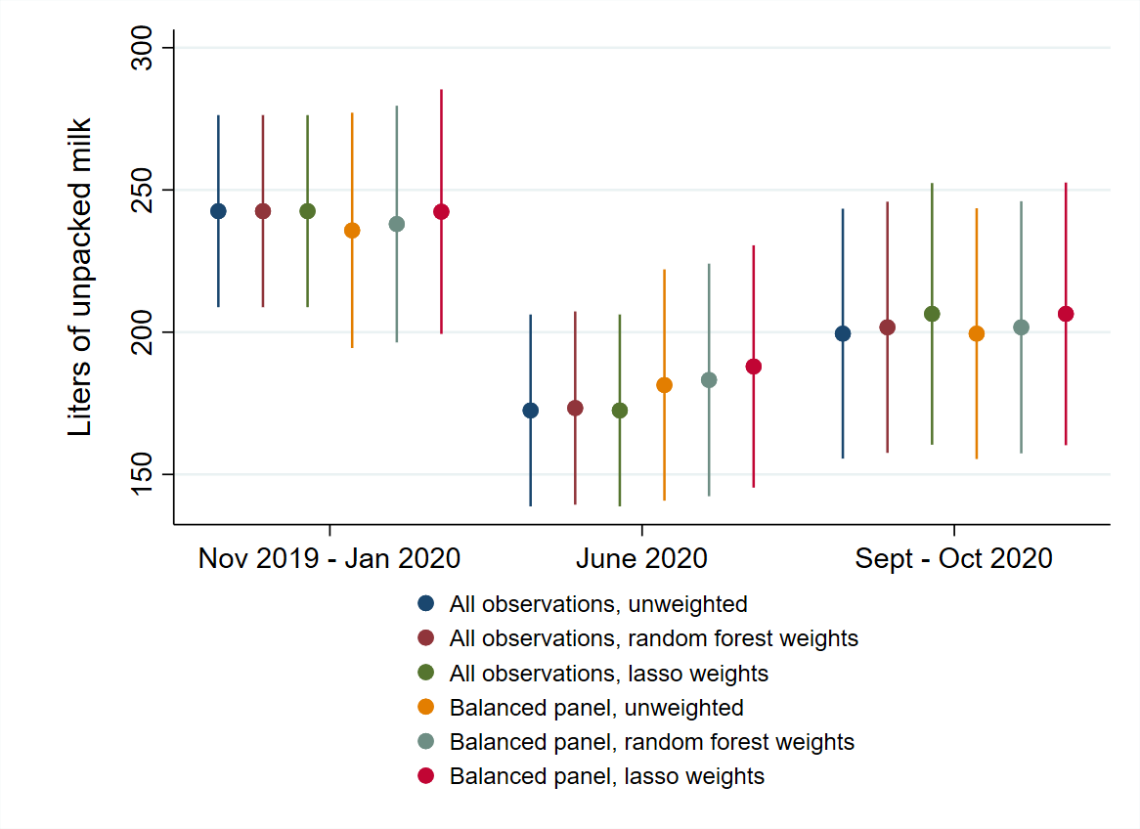


**Figure s3: Household dairy expenditures (Kenyan shillings), per adult equivalent, in past 30 days**


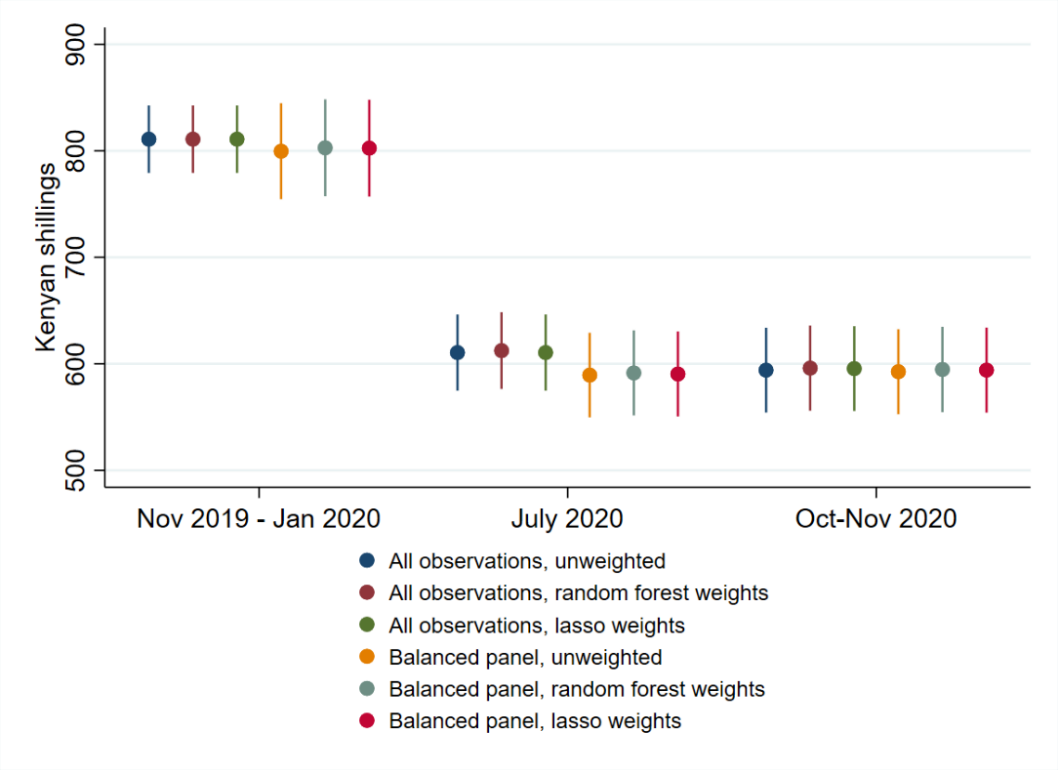


**Figure s4: Household food insecurity and access score**


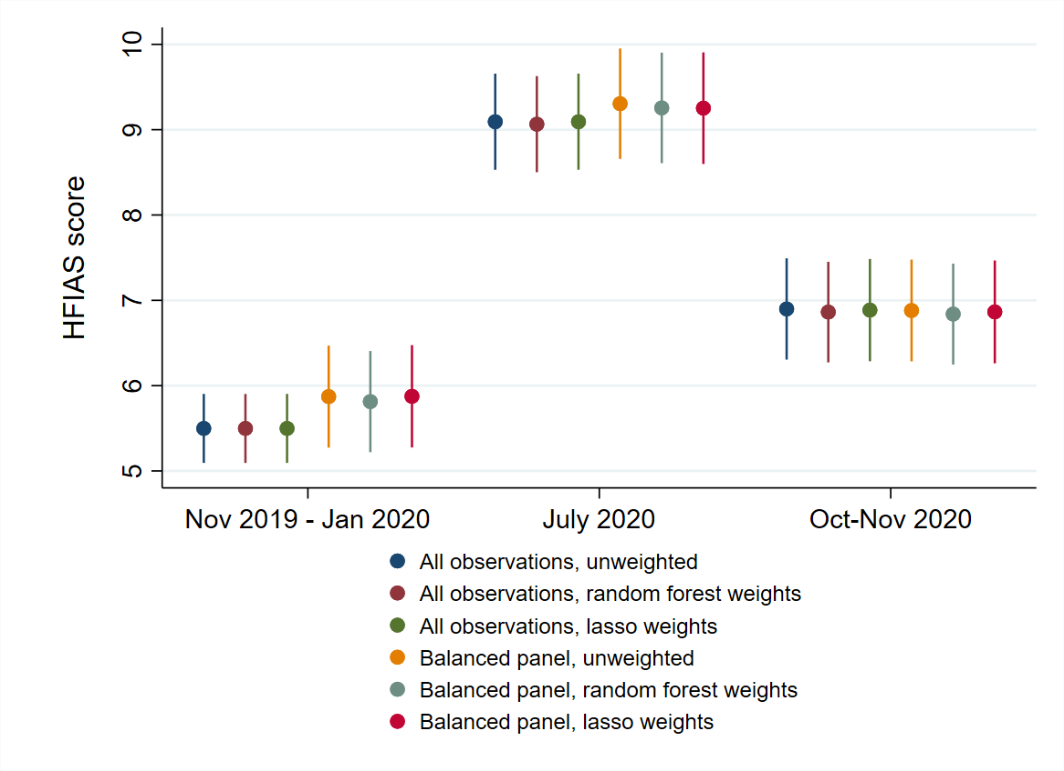

Supplement: Multimedia component 1 [file mmc1.docx]
