# Supplementary material for: Consumer Demand for Milk and the Informal Dairy Sector Amidst COVID-19 in Nairobi, Kenya
Source: Curr Dev Nutr. 2023 Feb 25;7(4):100058. doi: 10.1016/j.cdnut.2023.100058 (PMC9957657; doi:10.1016/j.cdnut.2023.100058)
Supplement: Multimedia component 2 [file mmc2.docx]

**Table s1: Vendor outcomes: unadjusted changes over time**

|  | **Baseline** | | | **Follow-up 1** | | | **Follow-up 2** | | |
| --- | --- | --- | --- | --- | --- | --- | --- | --- | --- |
|  | **N** | **% or mean ± SD** | **Median (IQR)** | **N** | **% or mean ± SD** | **Median (IQR)** | **N** | **% or mean ± SD** | **Median (IQR)** |
| Unpacked milk purchases, past 7 days |  |  |  |  |  |  |  |  |  |
| Total volume, L | 236 | 240.2±286.3 | 140.0 (70.0-300.0) | 199 | 169.7±213.5 | 70.0 (35.0-225.0) | 159 | 200.6±281.4 | 105.0 (35.0-245.0) |
| Average price sourced from suppliers, USD/L | 226 | 0.49±0.06 | 0.49 (0.44-0.52) | 167 | 0.49±0.04 | 0.49 (0.49-0.51) | 138 | 0.49±0.07 | 0.49 (0.49-0.52) |
| Unpacked milk sales, past 7 days |  |  |  |  |  |  |  |  |  |
| Vendor selling, % | 239 | 99.6 |  | 199 | 84.9 |  | 159 | 86.8 |  |
| Total volume, L | 237 | 242.5±263.8 | 140.0 (70.0-300.0) | 175 | 172.5±226.6 | 70.0 (30.0-245.0) | 159 | 199.5±281.4 | 105.0 (35.0-245.0) |
| Average price sold, USD/L | 206 | 0.59±0.06 | 0.59 (0.59-0.59) | 145 | 0.59±0.06 | 0.59 (0.59-0.59) | 138 | 0.60±0.05 | 0.59 (0.59-0.59) |

**Table s2: Household outcomes: unadjusted changes over time**

|  | **Baseline** | | **Follow-up 1** | | **Follow-up 2** | |
| --- | --- | --- | --- | --- | --- | --- |
|  | **% or mean ± SD** | **Median (IQR)** | **% or mean ± SD** | **Median (IQR)** | **% or mean ± SD** | **Median (IQR)** |
| ***N*** | ***670*** | | ***450*** | | ***336*** | |
| **Food insecurity** |  |  |  |  |  |  |
| HFIAS score | 5.5±5.3 |  | 9.1±6.1 |  | 6.9±5.5 |  |
| Prevalence of food insecurity, % | 70.0 |  | 86.2 |  | 81.5 |  |
| **Staple consumption** |  |  |  |  |  |  |
| Monthly per AE maize flour consumption, g | 4979.9±2511.0 | 4292.9 (3244.7-6446.5) | 5435.8±3202.5 | 4732.9 (3189.4-6949.8) | 5094.4±3071.2 | 4203.2 (2920.0-6739.2) |
| Monthly per AE maize flour consumption, USD | 3.25±1.71 | 2.79 (2.08-4.21) | 3.44±2.32 | 2.81 (1.93-4.42) | 3.11±1.87 | 2.74 (1.76-4.01) |
| **Vegetables** |  |  |  |  |  |  |
| Monthly per AE tomato consumption, g | 1926.8±1082.3 | 1747.1 (1184.5-2537.3) | 1825.0±1102.6 | 1651.8 (1019.9-2400.0) | 1886.1±1095.0 | 1664.8 (1095.9-2497.2) |
| Monthly per AE tomato consumption, USD | 2.29±1.44 | 2.10 (1.21-2.85) | 1.63±1.03 | 1.40 (0.91-2.16) | 1.48±0.96 | 1.33 (0.75-2.03) |
| Monthly per AE onion consumption, g | 3079.8±1604.0 | 2617.5 (1967.2-4086.2) | 2539.3±1745.7 | 2075.1 (1444.1-3472.1) | 2810.3±1582.5 | 2458.0 (1724.3-3865.0) |
| Monthly per AE onion consumption, USD | 0.91±0.52 | 0.77 (0.56-1.17) | 0.92±0.67 | 0.73 (0.48-1.23) | 0.81±0.50 | 0.70 (0.47-1.10) |
| **Animal source foods** |  |  |  |  |  |  |
| Monthly per AE beef consumption, g | 832.3±905.1 | 655.1 (0.0-1233.3) | 662.9±836.9 | 403.1 (0.0-1008.4) | 646.7±798.9 | 399.3 (0.0-1016.4) |
| Monthly per AE beef consumption, USD | 3.27±3.51 | 2.72 (0.00-4.85) | 2.58±3.19 | 1.60 (0.00-3.91) | 2.61±3.29 | 1.63 (0.00-4.11) |
| Monthly per AE egg consumption, g | 370.6±429.7 | 297.0 (0.0-547.2) | 311.2±353.8 | 259.6 (0.0-460.6) | 312.7±365.6 | 256.6 (0.0-508.0) |
| Monthly per AE egg consumption, USD | 0.77±0.90 | 0.58 (0.00-1.17) | 0.68±0.77 | 0.57 (0.00-1.03) | 0.70±0.83 | 0.55 (0.00-1.11) |
| **Dairy** |  |  |  |  |  |  |
| Daily per AE unpacked milk consumption, ml | 329.2±170.6 | 300.5 (206.5-418.4) | 250.1±177.7 | 228.4 (109.9-363.6) | 238.7±149.3 | 229.0 (123.8-351.6) |
| Monthly per AE unpacked milk consumption, USD | 5.88±2.95 | 5.55 (3.94-7.37) | 4.57±3.01 | 4.18 (2.44-6.49) | 4.42±2.62 | 4.18 (2.59-6.47) |
| Monthly per AE dairy consumption, USD | 7.95±4.10 | 7.21 (5.00-9.94) | 5.99±3.79 | 5.32 (3.16-8.40) | 5.82±3.65 | 5.31 (3.21-7.70) |
| Daily per AE energy from dairy, kcal | 284.3±141.0 | 258.4 (181.8-357.0) | 217.7±143.6 | 191.5 (117.7-306.3) | 207.0±121.6 | 194.3 (121.7-289.1) |
| Daily per AE protein from dairy, g | 13.1±6.4 | 12.1 (8.4-16.4) | 10.1±6.5 | 9.0 (5.5-14.2) | 9.6±5.6 | 9.2 (5.6-13.3) |
| Daily per AE calcium from dairy, mg | 477.4±235.3 | 435.3 (308.9-602.0) | 368.0±243.7 | 324.4 (199.8-521.6) | 350.9±206.6 | 330.1 (206.8-486.9) |
| **Other food** |  |  |  |  |  |  |
| Expended/consumed food away from home in the past week, % | 38.1 |  | 20.9 |  | 28.9 |  |
| Expended/consumed soda in the past week, % | 25.2 |  | 13.8 |  | 17.0 |  |

**Table s3: Vendors reporting difficulties sourcing milk and reasons for these difficulties**

|  | **Follow-up 1** | |  | **Follow-up 2** |
| --- | --- | --- | --- | --- |
|  | **N** | **% or mean ± SD** | **N** | **% or mean ± SD** |
| Vendors reporting difficulty sourcing milk compared to last year, % | 146 | 34.2 | 138 | 39.1 |
| Reasons why sourcing milk is more difficult | 50 | 1.7±0.9 | 54 | 1.3±0.7 |
| Less milk availability, % | 50 | 40.0 | 54 | 87.0 |
| Difficult transport, % | 50 | 44.0 | 54 | 5.6 |
| Expensive supply, % | 50 | 6.0 | 54 | 7.4 |
| Less cash/credit, % | 50 | 12.0 | 54 | 9.3 |
| Health concern (getting infected), % | 50 | 14.0 | 54 | 3.7 |
| Family demands (health-related), % | 50 | 0.0 | 54 | 1.9 |
| Family demands (non-health-related), % | 50 | 0.0 | 54 | 0.0 |
| Business operations restrictions, % | 50 | 46.0 | 54 | 9.3 |
| Moved out of Nairobi, % | 50 | 2.0 | 54 | 0.0 |
| Other, % | 50 | 0.1 | 54 | 0.1 |
